# Mind Over Medical School: A QIP on Wellbeing Interventions for Medical Students on Their Psychiatry Rotation

**DOI:** 10.1192/bjo.2025.10364

**Published:** 2025-06-20

**Authors:** Azjad Elmubarak, Sian Davies, Katie Dichard-Head, Ahmad Mohamed Kamal, Roshni Bahri

**Affiliations:** Birmingham and Solihull Mental Health NHS Foundation Trust, Birmingham, United Kingdom

## Abstract

**Aims:** The mental wellbeing of medical students has remained a pressing issue. A recent longitudinal study named ‘less supportive’ educational environments as a contributing factor to this ill-health. Anecdotally, authors of this study have found topics taught within psychiatry can be emotionally affronting for students. During their psychiatry placement, 4th-year medical students at the University of Birmingham and Aston University were offered voluntary interventions with the aim to foster an environment of wellbeing. These included 1) an Open-Door Policy with Clinical Teaching Fellows (CTFs), 2) a formal Drop-in Session, 3) a Psychiatry Film Club Evening, and 4) a Creativity Prize, for students to submit reflective pieces in any artistic medium. A mandatory final wellbeing lecture included personal testimony from two CTFs on their own mental health journeys.

**Methods:** All students were asked to complete pre- and post-placement questionnaires accessed online on their first and last day, no matter their participation with interventions. During the placement, interventions were promoted after plenary lectures and on an ad-hoc basis. The post-placement questionnaire ascertained student participation in interventions. Questionnaires used a forced Likert scale to measure agreement with various statements. Statements were developed by adapting validated tools (such as ATP-30 and MICA-4) to cover three domains: perceptions of psychiatry’s culture of wellbeing; stigma toward others’ mental health; stigma toward one’s own mental health. 117 responses were gathered. All responses were anonymous and could not be linked to individual students.

**Results:** Of the 177 respondents: 99% attended the mandatory wellbeing lecture, 11% attended the formal CTF drop-in, 9% participated in the creativity prize, 7% joined the film club, and 3% used the informal open-door policy. Across all domains, there was a general shift toward more favourable perceptions. Notably, responses to the statement “Psychiatry prioritises the wellbeing of its clinicians” improved from a median of “agree” to “strongly agree”. This was a statistically significant change. Stigma toward personal and colleagues’ mental health remained more resistant to change.

**Conclusion:** Results suggest that these interventions had a meaningful impact on students’ perceptions of psychiatry as a supportive specialty. Aside from obvious personal benefit, integrating wellbeing initiatives into clinical placements may be key in promoting psychiatry as a speciality to medical students. Larger sample sizes and additional data collection may be needed to detect more nuanced effects of these interventions: particularly in areas concerning self-stigma. Incorporating free-text responses in future evaluations could provide valuable qualitative insights into students’ experiences.

